# A Qualitative study of language barriers between South African health care providers and cross-border migrants

**DOI:** 10.1186/s12913-017-2042-5

**Published:** 2017-01-31

**Authors:** Jo Hunter-Adams, Hanna-Andrea Rother

**Affiliations:** 1 Health Economics Unit, School of Public Health and Family Medicine, University of Cape Town, Anzio Rd, Observatory, 7925 South Africa; 2 Environmental Health Division, School of Public Health and Family Medicine, University of Cape Town, Anzio Rd, Observatory, 7975 South Africa

**Keywords:** Language, Refugee health, Migrant health, Health care access, Medical interpretation, South Africa

## Abstract

**Background:**

Communication with health care providers represents an essential part of access to health care for the over 230 million cross-border migrants around the world. In this article, we explore the complexity of health communication from the perspective of cross-border migrants seeking antenatal care in Cape Town, South Africa in order to highlight the importance of high quality medical interpretation.

**Methods:**

As part of a broader study of migrant maternal and infant nutrition, we conducted a secondary data analysis of semi-structured in-depth interviews (*N* = 23) with Congolese (*n* = 7), Somali (*n* = 8) and Zimbabwean (*n* = 8) women living in Cape Town, as well as nine focus group discussions (including men: *n* = 3 and women: *n* = 6) were conducted with migrant Somalis, Congolese, and Zimbabweans (*N* = 48). We first used content analysis to gather all data related to language and communication. We then analysed this data thematically.

**Results:**

Zimbabwean participants described how the inability to speak the local South African language (IsiXhosa) gave rise to labelling and stereotyping by healthcare staff. Congolese and Somali participants described medical procedures, including tubal ligation, which were performed without consent. Partners often tried to play the role of interpreter, which resulted in loss of income and non-professional medical interpretation. Participants’ highlighted fears over unwanted procedures or being unable to access care. Challenges of communication without a common language (and without professional medical interpretation), rather than outright denial of care by healthcare professionals, mediated these encounters.

**Conclusion:**

Although there are several factors impeding cross-border migrants’ access to health care, effective communication is a prerequisite for quality care. Free-to-patient professional medical interpretation would not only benefit migrant populations but would benefit the broader community where language and health literacy are barriers to accessing health care. Novel approaches to language access may include technology-enabled professional interpretation.

## Background

In 2013, there were more than 232 million cross-border migrants globally [1]. Cross-border migrants have heterogeneous life experiences and travel for many different reasons. They may have experienced discrimination and marginality before, during, or after travel. Given the magnitude of international movement, facilitating healthy migration and providing high quality care to migrant populations needs to feature clearly in health policy and health systems. We focus on the challenge of providing quality care in the context of migration between low-and-middle-income countries, using South Africa as an example. While the notion of quality care has many facets, this article focuses on communication as an essential precursor for many aspects of health care quality, including considering medical advice and taking medicine correctly. Communication across language and cultural barriers has received much interest in certain settings, notably in the U.S. However, in low- and middle-income (LMIC) settings health communication tends to have low priority relative to other pressing issues in the health system. In this article we attempt to document the importance of communication for migrants seeking maternal care in Cape Town, South Africa.

Given stretched public health services, providing adequate access to health care services for all is a current challenge in South Africa. Migrants, however, face particular challenges that are often exacerbated in relation to accessing services. The South African constitution [2], the South African Refugees Act [3] and the right to basic health care (Section 27 (1) (a)) present ambitious standards for realising human rights and the dignity of all, including all migrant groups. However, in many cases government fails to provide services that live up to these standards for the general population. While recognizing that both migrants and South Africans face a complex slew of challenges in accessing health services (e.g., long wait times, medication shortages), in this paper we argue that language is a key obstacle to adequate medical care and health literacy. Particularly, we assess the impact of language barriers for cross-border migrants on accessing care, and understanding the implications of medical treatments recommended.

Post-apartheid, South Africa is an important destination country for African migrants seeking work and/or asylum. In Cape Town, cross-border migrants constitute at least 3% of the population, and may make up as much as 9%, due to large numbers of individuals’ country of birth being listed as “unspecified” [4]. Yet cross-border migrants in South Africa continue to be marginalized and subject to a broad range of discrimination (e.g. violence against foreign-owned businesses, job discrimination) [5, 6]. Experiences of exclusion are exacerbated by lack of legal status, as well as minority status relative to South African population groups. Despite evidence that urban migrants actually contribute to the economies of their new cities [7, 8], cross-border migrants in South Africa continue to be portrayed as placing a burden upon the public healthcare system [9]. As Crush and Tawodzera [10] rightly point out from their research, xenophobia in South Africa can also be witnessed in the public health system which they refer to as “medical xenophobia”. Examples of this include hospital security declining non-nationals entrance into a health facility, and health professionals placing non-nationals in a longer queue [10]. Language barriers and the lack of medical interpretation are further examples where migrants experience “medical xenophobia”.

## Methods

Fieldwork for this qualitative study was conducted between February and October 2013, and the findings presented in this paper are based on language issues raised during in-depth interviews and focus group discussions. The results presented in this paper are derived from a broader study of cross-border migrants experiences of maternal and infant nutrition in Cape Town, South Africa, where language and communication emerged as an unintended theme [11, 12]. We hold to the constructionist tradition [13] that meaning and analyses are constructed rather than fixed, and this analysis represents a contribution to our understanding of patient experiences of multilingual medical encounters in a specific context of cross-border migration to a LMIC.

### Study setting

The study was conducted across greater Cape Town. Cape Town is in the Western Cape province of South Africa where 28% of residents are not born in the province and up to 9% are born in another country [4]. The participants in this sample predominantly resided in rooms with their partner and children, sharing amenities with other families, either in a larger house or apartment, or in illegally subdivided warehouses. Most interviews took place in migrant homes, whereas focus groups took place primarily in more communal settings, including community centres, shops, and a women’s shelter. While one focus group took place with residents of an informal settlement with shack housing, participants more commonly resided in inner city settings across many parts of Cape Town. Although the participants in this sample largely occupied these relatively crowded inner city spaces, it should be noted that cross-border migrants in Cape Town have a wide range of educational and socio-economic backgrounds, and have a broad range of professional roles in South Africa. The experiences of participants in this sample were thus grounded in a specific socio-economic context, in particular, of life in the inner city, and to a lesser extent, in informal settlements.

### Study participants and sampling

Study participants were selected to participate either in in-depth interviews or in a focus group. Participants had typically resided in South Africa for less than 10 years. For the in-depth interviews, 23 Somali (*n* = 8), Congolese (*n* = 8), and Zimbabwean (*n* = 7) women were purposively selected to include different migrant groups who could provide diverse insights. The interview inclusion criteria was: women over the age of 18 who were pregnant or had given birth in the last 2 years, and self-identified as Somali, Congolese (from the Democratic Republic of Congo, DRC), or Zimbabwean. The majority of in-depth interview participants were married. Nine separate focus group discussions (*N* = 48) where held with adult Somali, Congolese, and Zimbabwean men (*N* = 3; *n* =21) and women (*N* = 6; *n* = 27), segregated by country of origin and gender. In the parent study, in-depth interviews were used to attain depth of knowledge about individual perceptions of maternal and infant nutrition, while focus groups provided perspectives on collective meanings and understandings—the collective narrative [14]—related to maternal and infant nutrition in Cape Town. Both in-depth interviews and focus groups consisted of a convenience sample using snowball sampling, an effective technique among hard-to-reach populations [15], and used both non-governmental organization (NGO) contacts and individual introductions to begin meetings with eligible cross-border migrant participants. These NGOs worked across different parts of the city, with various populations. Including both in-depth and focus group discussions, there were 26 Congolese participants (16 women, 10 men), 21 Zimbabwean participants (16 women, 5 men) and 24 Somali participants (18 women, 6 men). More women than men were sampled due to the original study design which focused on maternal and infant nutrition. This may mean that more language issues were described, as women were less likely to have paid employment or to have had other opportunities to become conversant in English. Nevertheless, maternal care is also one of the key points of care for migrants, and thus language in the specific context of maternal care is an important issue for migrant health [16, 17].

### Data collection

JH-A conducted all the interviews and focus groups. Two focus group discussions, four Somali in-depth interviews and one Congolese in-depth interview also included a trained interpreter to assist JH-A. In-depth interviews lasted between 1 and 1.5 h, and were primarily conducted in participant homes. Focus group discussions took place in community centres and lasted between 1.5 and 2 h.

The objective of the broader study was to garner meanings and understandings and dominant discourses related to maternal and infant nutrition in a migrant context. An interview guide framed the semi-structured in-depth interviews [12]. When participants discussed health care it was frequently in relation to accessing healthcare during pregnancy, and how participants felt this related to feeding decisions. Questions in focus groups broadly related to comparing experiences of food provision and nutrition advice for pregnant mothers and new babies living in South Africa versus practices in participants’ countries of origin. In this paper, the focus is on participants’ unprompted discussions that related to language and healthcare provision in Cape Town public hospitals, as well as primary health care clinics.

#### Use of language interpretation in data collection

Our use of interpretation in this study has been briefly described in a previous publication [12]. Nevertheless, it is important to discuss in more detail issues of meaning and language related to the our research process, to make the multiple languages used in the study more visible and consider the implications related to conducting a study in multiple languages [18]. Interpretation was not clean-cut; in Cape Town there are multiple South African languages spoken by residents. Amongst Congolese migrants, participants often used multiple languages in a single conversation. As such, making meaning, and settling on specific choices of words in transcripts, involved much negotiation [19]. This negotiation began with the process of simultaneous interpretation, and weighing how to manage interviews alongside interpreters, who were situated as a type of bridge between the participant and the interviewer. In in-depth interviews, we found that trying to make interpretation overly formal circumscribed free conversation, while allowing the interpreter to be a third party in the conversation, made the participant more comfortable. Larkin and colleagues [20] termed this process “mutual reciprocity”. Zimbabwean participants were comfortable using English in the interview setting, though they may speak English, Shona, and/or Ndebele amongst family and friends. Somali participants typically preferred to speak Somali, whereas Congolese participants preferred a mixture of languages (French and either Swahili or Lingala) or had become fluent in English through work or studies in Cape Town. Independent professional medical interpreters then checked the quality of interpretation (comparing audio to English transcripts), discussed the role of the interpreter, and discrepancies and alternative meanings with the moderator/interviewer (JHA). Transcripts were then revised and discussed with the independent professional interpreter to enhance the quality of the final “verbatim” transcript. We recognize that the process of speaking on behalf of any participant group, particularly when engaging in research, presents potential research limitations.

#### Data analysis

In this paper we focuses specifically on categories related to language and communication. These categories were identified during overall data analysis of the broader research question. The broader data analysis was guided by principles of thematic analysis [21, 22]. This analysis began during fieldwork in the form of a research diary, notes, and reflections. After immersion in both focus group and interview transcripts through reading and rereading, an inductive initial analysis was used to generate a codebook and code all transcripts [23]. After developing and defining an initial set of codes, we began to code transcripts and test validity of the codes and the extent to which they seemed to convey the meanings and understandings presented by participants. Once no new categories of codes appeared, the codebook was considered complete and coding of all transcripts- both in-depth interviews and focus groups- were coded using the complete codebook. All transcripts were uploaded to the computer software Hyperresearch (Researchware Inc., 2009, Massachusetts, U.S.A.), to assist with coding, sorting, and data management.

Based on our analysis, language and communication in health care settings represented a key theme through which participants understood their experience of maternal and infant nutrition in Cape Town. As such, we performed additional content analysis [24] to focus specifically on instances involving language barriers. This represented a secondary data analysis design, and was conducted by the same researchers who designed, implemented, analysed, and reported on the primary data analysis [25, 26].

## Results

We frame our results around the fundamental issue of the language barrier resulting from the lack of a common language. This was by no means the only issue with accessing health care, but it was among the significant reasons that participants felt unable to get adequate care. The issue of language emerged as an unexpected result in the context of broader questions related to maternal and infant nutrition. Comments related to language were raised whenever participants spoke of South African health services. As healthcare services represented a relatively minor line of questioning in interviews, language discordance was raised an issue explicitly in a small number of in-depth interviews. However, it was also raised frequently the nine focus group discussions, where language and lack of communication evoked very strong feelings and was often spoken about spontaneously. As an important topic for participants, we felt that language and communication deserved further analysis.

### Lack of a common language: Refusal to speak English

While reports of nurse abuse of patients is not specific to cross-border migrants [16, 17], participants’ perception of language as a vehicle of discrimination was exacerbated by participants’ experiences of discrimination outside the healthcare system. For example, among Zimbabweans, participants’ interpreted the language barrier as an unnecessary imposition: Nurses could speak English but tended to communicate in IsiXhosa, despite the fact that Zimbabweans could not understand this South African language:

When we go to the clinics for the check-ups, ne, some of the nurses they are Xhosa some are coloured,^1^ they are different, especially if we meet‚ the Xhosa nurses‚ sometimes they can speak to them [Zimbabweans] in their language, of which we don’t understand their language. If you speak to them in English they can ignore you. Or, they can shout at you. It is bad for us. Of course, just because we don’t understand their language. (Zimbabwean Women’s Focus Group)

The use of isiXhosa by medical support staff was also perceived as a vehicle whereby Zimbabwean participants felt they were discriminated against. For example, study participants described calling for an ambulance and it not showing up, and feeling that if:

You can give the Xhosa [person] the phone, to phone, right there the ambulance would come. Within 20, 30 minutes the ambulance would come…[…] but [for us] the ambulance didn’t come. Until when I reached here, I found that the baby was delivered here in the house. Ja. So it’s very hard. (Zimbabwean Women’s Focus Group)

Moreover, issues of language were raised in conjunction with challenges of being and feeling alone in Cape Town. The need for nurse advice was expressed as particularly important in the context of the social isolation experienced by young mothers.

If she’s speaking in Xhosa, and she is giving me advice, how can I understand? When I don’t speak Xhosa. How can I know how to raise that child, when I don’t know!? Like, when it’s a small child [young mother], 17, 20, it’s your first child?! Like back home, there will be a big person, who will be teaching you how to bath the child, how to feed the child… But here‚ it’s nurses who are going to teach you. (Zimbabwean Women’s Focus Group)

Participants seldom referred to high-level providers, such as doctors (who may be more likely to communicate with them in English), and much more on the roles of lower and mid-level providers such as nurses, front-desk receptionists and similar “gatekeepers”. For Zimbabweans, the fact that these staff spoke to them in Xhosa meant that they felt discriminated against by the healthcare system, unable to receive emergency care (ambulances), and unable to receive medical advice.

### Lack of a common language: Preventing informed consent

Participants largely did not describe being turned away from antenatal care, yet where migrants had strongly negative perceptions of health care, these perceptions revolved around being forced to receive interventions that they did not consent to. Similar experiences were recounted by many participants, and seemed to colour their experiences of health care in South Africa. Language barriers were a major feature in these descriptions:

I get good advice and bad advice here (laughter). Is another people, you know we come from French place, we didn’t come from English place… we come from a French place. Now when you come here your English is too small, you don’t know anything when you go to the hospital. The doctor… you’re pregnant… you go to the hospital, […] they just ask you, you want to cut, you want to make family planning, you want to cut your tubes and you don’t know what they are talking, you just say yes, but if you have paper you sign, you go. (Congolese mother of six children)

Multiple Somali participants-- both in the men’s focus group and in individual interviews-- described cases in which Somali women were sterilized without consent. This story had reverberated throughout the community, leading women to be very fearful of and mistrust healthcare providers.

They came to her when she was sick, and she had no husband there, and told her to sign… and she had no idea what she was signing. And they did the process [tubal ligation]… and she can’t deliver anymore, even permanently. So when they go there, to the hospital, to complain, they said “you signed”, but she didn’t understand, and she was sick. That’s why I’m concerned. That’s why I’m very concerned. (Pregnant Somali mother of five children, Interpreter present)

While health care providers may perceive an offer of family planning advice as standard procedure, the lack of medical interpretation, together with women’s marginality in Cape Town, meant that women felt coerced to receive interventions, or even that they received an intervention without knowing. These interventions, and the ways they were interpreted, take place against a backdrop of staff shortages and xenophobic violence in South Africa. However, without a means of communication, stereotypes and misunderstandings could not be challenged, resolved or discussed. For all three population groups, participants’ experiences translated to an overall lack of trust in the healthcare system. In turn, this limited their ability and willingness to seek out advice of healthcare professionals more generally, and made them more likely to distrust whatever advice they did receive.

We highlight the experiences of one particular participant to illustrate the specific cascade of events that can occur when communication between provider and patient is inadequate. Halima^2^ was a 26-year old Somalia pregnant mother of five who could not speak English. She described suffering from high blood pressure and gestational diabetes during previous pregnancies. Halima’s first husband had been murdered in the South African xenophobic attacks of 2008. Approximately 6 months pregnant, she had not yet received any antenatal care fearing that she would be shouted at for becoming pregnant (a common occurrence described by Somali participants). During a previous pregnancy, she described being cautioned that if she returned pregnant, she would have a procedure to prevent her from becoming pregnant again.

I gave birth to some of my children at [hospital name], some at [hospital name], but the last time they told me, no this time, they gonna sew… something like tubes… I don’t know… I am thinking of maybe going to [alternative hospital], because … [but] they might do that. Stop me for 5 years… and that’s something I’m against. So I’m thinking of private hospitals, but I’m also thinking, it’s too expensive! (Pregnant Somali mother of five children, Interpreter present)

Describing a deeply antagonistic relationship with health care providers and concerned about stories of other Somali women who had reportedly undergone forced sterilisation, Halima was potentially putting herself at risk of health complications. She was alone with her children during the week as her husband worked running a small shop in an informal settlement. In previous pregnancies she had to go alone to medical appointments, and she was unable to speak English.

Halima describes “healer shopping” [27], where, finding it impossible to communicate her priorities and choices to providers, she started shopping around for a hospital that would not “know” her. She felt as though she had been blacklisted and was being punished for having children, rather than feeling that the health system wanted the best outcome for her. These fears were amplified by the stories of forced sterilizations that circulated within multiple migrant communities, including Somalis and Congolese. It was apparent that she and providers had to somehow communicate largely without language, and that the two-way communication necessary to convey health information as well as patients’ concerns and interests, was lacking. From Halima’s perspective, xenophobic health care providers did not want her to have children, and did not respect Somalis’ practice of Islam, which precluded family planning except in the case of serious illness. Without communication between medical provider and patient, there was no opportunity for Halima to understand the risks related to high blood pressure and previous gestational diabetes, and in becoming pregnant again and arriving for antenatal care very late in pregnancy, it seemed likely that the negative cycle between provider and patient would be further entrenched.

### Lack of a common language: Migrant responses

During all three men’s focus group discussions, participants’ described accompanying their wives and partners to appointments to interpret for them. Descriptions by English-speaking Congolese and Somali men revealed some of the complexity of juggling appointments, particularly in the context of work.

Assisting her because she didn’t know English as well and she has to go, before she goes to hospital I have to write everything in a paper and when you go you have to say—give them the paper, he will read the paper. So when they do all the tests then I ask her also to ask the nurse to write in the paper so I can understand and explain to her at home […] I don’t have anyone here like to assist and take her to hospital and I had a tough time at work as well because sometimes I have to excuse myself from work and take her to hospital, even when the child was born, ja, those are kind of difficulties we experience. (Congolese Men’s Focus Group)

It is common for family members to be used as *ad hoc* interpreters in the South African healthcare system, both for South Africans and non-South Africans. Whereas for South Africans, children, cleaning or health care staff are often described as playing the role of interpreter [28], for non-South Africans in this study, this role was placed on the male partner. This role was in turn viewed as a “burden” as expressed as a dominant theme in the Congolese and Somali focus groups.

## Discussion

While much has been written about medical interpretation in high-income countries (HIC) [29–31], most notably in North America, South Africa is one of the only low and middle-income countries (LMIC) where studies of medical interpretation have taken place. Previously, the experiences of non-English speaking South Africans have been highlighted in the South African context, largely in urban settings such as Cape Town [28, 32, 33]. In South Africa, there is currently no official system for medical interpretation (where medical interpretation is the facilitation of communication between a medical provider and patient who do not speak the same language). Given that South Africa is a multilingual society with 11 official languages and many South Africans do not speak English, up to 80% of health care consultations in South Africa are carried out across linguistic and cultural barriers [34], and the language barrier has become normative and even invisible [28]. Yet the consequences of the language barrier has been to essentially “silenc[e] the patient’s voice” (p58) [27]. It compromises patient quality of care, where those who cannot communicate with health care providers are less likely to adhere to treatment, to seek care or follow-up appointments, or receive preventive services [33].

The lack of professional medical interpretation affects adequate health care provision. *Ad hoc* interpretation or no interpretation results in misdiagnosis and the inability to understand or follow medical recommendations [27]. Interpretation by male partners in the case of cross-border migrants placed a financial burden on families who were made all the more vulnerable by spouses taking time off work. Where husbands were positioned as gatekeepers for their spouses’ care, women were dependent and vulnerable. Moreover, the use of partners as *ad hoc* interpreters violated confidentiality and increased the likelihood of medical error. In one study based in the U.S., the use of *ad hoc* interpreters actually resulted in poorer communication than no interpretation whatsoever [35]. Whereas professional medical interpretation has been presented as prohibitively expensive, these costs must be weighed against the economic and other costs of medical error as well as the costs of *ad hoc* partner interpretation to an already financially marginal family unit. Medical interpretation needs to feature in a broader measure to improve health literacy among all living in South Africa.

Our study is suggestive of the experience of language discordance as a key hurdle impeding medical care between migrants and health care providers. The language barrier, however, is symbolic of deeper perceptions and attitudes of lack of trust. To the extent that the language barrier was an intentional imposition for English-speaking Zimbabweans, it presents an example of xenophobia, and adds to existing evidence of this phenomenon. In other cases, particularly for non-English speaking Congolese and Somali participants, the consequences of lack of a common language included lack of consent for medical procedures. This was closely linked to a lack of trust, resulting in a patient opting out of much-needed medical care. Lastly, the inability of cross-border migrants to communicate with health care providers led to untenable financial costs to the household in cases where family members were forced to miss work to accompany partners to appointments. In the long run, therefore, the lack of medical interpretation in this population could clearly affect the health outcomes of the patient, as well as the long-term medical costs of late entry into care, lack of ability to communicate symptoms, and lack of ability to follow medical advice.

The findings of this study demonstrate ways that professional medical interpretation may be a vital part of access to health care in cases where patient and provider lack a common language. The mismatch between the strongly worded human rights orientation of the South African constitution and the lack of capacity at a health systems level speaks to a core challenge of the South African health system in living up to the standards of the constitution. However, given that human rights motivation for medical interpretation is largely established [32], the question becomes one of practical application and financing, as well as one of political will and prioritization of health needs.

One practical barrier to interpreter services in settings where there is precedent for providing professional services has long involved the question of who should pay for interpreter services, given stretched resources. While this question is a challenging one, it is important to note that there is evidence to suggest that providing interpreter services greatly increased uptake of preventive visits [30], and that the provision of interpreter services makes patient visits much more efficient, saving money in the long term [31]. In a study of interpretation in the United States, patients with low English proficiency (LEP) who did not receive professional medical interpretation were hospitalised for significantly longer and were significantly more likely to be readmitted within 30 days [29]. A second study found that LEP patients received significantly more preventative services, more office visits, had more prescriptions filled when they had professional medical interpretation during medical visits [30]. Moreover, the financial cost to families who provide *ad hoc* interpretation to spouses has ethical and financial costs, increasing the likelihood of medical error [35] and resulting in a potential loss of earnings. In the South African context, an argument can and should be made that increased costs related to medical interpretation may be offset by the lower costs of improved adherence, reduced hospital stays in the public health system, and ultimately, better health outcomes. Given that medical interpretation is a global issue, interpretation may involve collaboration between trained bilingual individuals in migrants’ country of origin. For example, the use of skype calls may be a potentially affordable means to facilitate communication in cases where there are few truly bilingual individuals in the new country.

Although medical interpretation represents an important step to improve health communication through addressing language barriers, it may not necessarily address the more deep-seated xenophobic attitudes. Who does the medical interpretation, however, must be considered so that trust of the interpretation is shared both on part of the migrant patient and the health care provider. Medical interpretation should therefore feature more prominently both as part of medical education and government health policies.

### Limitations of the study

This study represents the perspectives of purposively selected participants of three specific nationalities living in Cape Town although there are other migrant populations. As such, it is both population and geographically specific. Our methodology of secondary data analysis of in-depth interviews and focus groups also presents limitations to the broader applicability of our findings, as the data was derived from limited communication-related questions. Language barriers were not the specific focus of this study and so further probing and questioning was limited. That said, perspectives on language reached saturation, as diverse participants attending multiple health facilities expressed similar sentiments around lack of communication. Further studies of language discordance may require larger, more diverse samples in order to better understand the extent of this issue in South Africa, and elsewhere. Evaluation and discussion of existing language-based interventions in low- and middle-income settings would assist the development of appropriate responses.

### Strengths of the study

This study represented one of the first efforts to understand the experiences of cross-border migrants in healthcare settings in Cape Town. As a group that is potentially vulnerable to poor health care and poor health, given xenophobia, lack of legal status, as well as other factors, understanding these experiences represents an important first step in improving migrants’ health.

## Conclusions

While in this article we focused on the South African case, issues of communication between health care professionals and cross-border migrant patients are global. Free-to-patient medical interpretation is one key intervention to address language barrier between health care providers and patients. In South Africa, advocating for such a service on a wide scale may first need to establish two things. Firstly, advocates need to establish that interpretation improves health outcomes, which to an extent has already been established, both in South Africa [32], and elsewhere [30]. Secondly, it must be established that professional medical interpretation is practical and implementable at a health systems level. For this, cost-effectiveness studies may help to make the financial case for the importance and feasibility of professional medical interpretation in South Africa. Given the limitations of the South African public health system, and numerous competing priorities, implementation at a health systems level is challenging. Remote interpretation, either via phone or videoconferencing, may provide a cost-effective alternative to in-person interpretation, where language volume in a particular geographic location is low [36]. Such an approach may be feasible in urban contexts such as Cape Town. In South Africa, piloting medical interpretation with a specific cross-border migrant group where a clear need has been demonstrated, at one or two hospitals, could pave the way for interpretation for other migrant groups, as well as South Africans.

The broader challenge of xenophobia, both in medical settings and in communities, may partially be addressed by better communication, to the extent that xenophobia is rooted in ignorance, although xenophobia also represents a deeply rooted systemic challenge. However, there is also a need to sensitise health care providers and frontline staff to the needs of migrants, as well as to dispel negative stereotypes of cross-border migrants. Longer-term measures to address these through the medical education system and health care policies are needed.

## Abbreviations

HIC: High income countries
LMIC: Low and middle-income countries

## Footnotes

1 The term “coloured” has roots in apartheid racial categories, and refers to individuals of mixed ancestry, including European, African and Asian ancestry.

2 Not her real name
